# Sensitivity to Photoperiod Is a Complex Trait in *Camelina sativa*

**DOI:** 10.1002/pld3.70071

**Published:** 2025-04-15

**Authors:** Bryan A. Ramirez‐Corona, Erin Seagren, Carissa Sherman, Takato Imaizumi, Christine Queitsch, Josh Cuperus

**Affiliations:** ^1^ Department of Genome Sciences University of Washington Seattle Washington USA; ^2^ Department of Biology University of Washington Seattle Washington USA; ^3^ Brotman Baty Institute for Precision Medicine University of Washington Seattle Washington USA

**Keywords:** *Camelina sativa*, day neutrality, photoperiod response, photoperiod sensitivity, response to day length

## Abstract

Day neutrality, or insensitivity to photoperiod (day length), is an important domestication trait in many crop species. Although the oilseed crop *C. sativa* has been cultivated since the Neolithic era, day‐neutral accessions have yet to be described. We sought to leverage genetic diversity in existing germplasms to identify *C. sativa* accessions with low photoperiod sensitivity for future engineering of this trait. To do so, we quantified variation in hypocotyl length across 161 *C. sativa* accessions of 4‐day‐old seedlings grown in long‐day and short‐day conditions as a high‐throughput approximation of variation in the photoperiod response. Soil‐grown adult plants from selected accessions also showed variation in the response to day length in several traits; however, the responses in seedling and adult traits were not correlated, suggesting complex mechanistic underpinnings. Although RNA‐seq experiments of the reference accession Licalla identified several differentially regulated *Arabidopsis* syntelogs involved in photoperiod response and development, including *COL2*, *FT*, *LHY*, and *WOX4,* expression of these genes in the accessions did not correlate with differences in their photoperiod sensitivity. Taken together, we show that all tested accessions show some degree of photoperiod response and that this trait is likely complex, involving several and separable seedling and adult traits.

## 1 Introduction

Climate change, population growth, and the loss of arable land are major challenges that threaten food security. One approach to ensuring food security is the development of sustainable low‐resource crops that can grow on marginal land, are stress‐resistant, and are high‐yielding (Berti et al. 2016). One such crop is *C. sativa,* a low‐resource oilseed crop that is amenable to genetic engineering (Berti et al. 2016). Because of *C. sativa's* agricultural potential, recent studies have developed genetic resources, genome sequence, and expression data, among other resources for this crop (Kagale et al. 2016 *;* King et al. 2019; Luo et al. 2019; Gomez‐Cano et al. 2020).

*C. sativa* grows well in marginal soils, adapts readily to adverse environmental conditions, and has low water and nutrient requirements compared to other oilseed crops (Vollmann and Eynck 2015). Unlike the high‐yielding oilseed crop *Brassica napus* (canola), *C. sativa* is resistant to common *Brassicaceae* pests and pathogens (Séguin‐Swartz et al. 2009). Camelina seed oil content ranges from 36% to 47% by weight, with exceptionally high levels of essential and omega‐3 fatty acids, a profile broadly useful in food, animal feed, industrial bioproducts, and biofuel (Berti et al. 2016; Yuan and Li 2020). In field trials, the crop reduces weed biomass through the release of inhibitory chemical compounds, demonstrating its allelopathic potential (Ghidoli et al. 2023). Camelina is readily transformable using the floral dip method, enabling genetic engineering (Lu and Kang 2008). Its short life span (85–100 days) and ability to be planted and harvested using conventional equipment make field trials of engineered plants straightforward (Malik et al. 2018).

The major reason for *C. sativa's* displacement by canola is the crop's modest seed yield (Obour et al. 2015; Berti et al. 2016). In many domesticated crops such as canola, rice, maize, sorghum, potato, and others, the loss of the photoperiod response—the acquisition of day neutrality—is common and has allowed farming of these crops at higher latitudes (Doebley et al. 2006). As a long‐day (LD) plant, *C. sativa* flowers in the spring at higher latitudes, thereby accumulating comparatively little vegetative biomass to produce carbohydrates and ultimately seeds. In canola, vegetative biomass is the primary determinant of seed yield (Bennett et al. 2017; Zhang and Flottmann 2016; Chen et al. 2021), so it stands to reason that one way to increase *C. sativa* seed yield is to generate day‐neutral varieties. Thus, to engineer or breed *C. sativa* cultivars with higher seed yield, it is imperative to understand both the extent of natural variation of the photoperiod response in this crop and its genetic underpinnings.

Here, we quantified the photoperiod response across 161 diverse *C. sativa* accessions by recording hypocotyl length and germination rate of seedlings grown in LD and short‐day (SD) conditions. Although the response to photoperiod has been historically measured as the time to flower in long and short days, the genetic networks governing differences in hypocotyl elongation in short and long days and in time to flower are highly interlinked in *C. sativa's* close relative *Arabidopsis thaliana* (Huang et al. 2016; Kim et al. 2005; Mizoguchi et al. 2002; Ronald et al. 2024; Sellaro et al. 2012; Undurraga et al. 2012; Zagotta et al. 1996). Because hypocotyls of young seedlings are amenable to high‐throughput measurements with large numbers of replicates, we used this approach to categorize accessions as either low‐ or high‐responsive to day length, using Licalla as a reference accession. Eight accessions with low, medium, or high hypocotyl response to day length were selected for measurements of adult developmental traits associated with photoperiod responses. Seedling responses were not predictive of photoperiod responses in adult traits. Gene expression levels of four genes with roles in photoperiod response or development—*COL2*, *FT*, *LHY* and *WOX4*—did not explain the accession‐specific differences. In sum, *C. sativa* accessions show a range of photoperiod sensitivity; however, there is little correlation across different traits, suggesting complex mechanistic underpinnings.

## 2 Results

The photoperiod response is typically determined by measuring the time to flower in the commonly used conditions short and long days. Measuring this adult trait across many different accessions with enough replicates for rigorous statistical comparisons is labor‐ and space‐intensive. However, this adult trait is linked to the seedling trait hypocotyl length via the genetic networks associated with the plant circadian clock (Zagotta et al. 1996; Kim et al. 2005; Mizoguchi et al. 2002; Niwa et al. 2009; Nagel and Kay 2012). Notably, the domestication events that are associated with day neutrality in common crops such as rice, barley, and tomato also affect the circadian gene networks that are known to affect hypocotyl elongation in short and long days in *A. thaliana* (Comadran et al. 2012; Goretti et al. 2017; Liu et al. 2018; Matsubara et al. 2012; Soyk et al. 2017; Takahashi et al. 2009; Turner et al. 2005; Xue et al. 2008; Zhang et al. 2019; Zhang et al. 2018). Therefore, we used hypocotyl length measurements as a high‐throughput proxy to explore natural variation in the response to day length across the 161 Camelina accessions.

To quantify the phenotypic variation in hypocotyl response to day length, we grew 161 *C. sativa* accessions (Table S1) under LD or SD conditions and measured the hypocotyl length of 4‐day‐old seedlings (Table S1). Accessions were split into 13 experimental batches. Each batch consisted of 32 seedlings per photoperiod treatment for each accession. The reference accession Licalla (Gehringer et al. 2006) included in each experimental batch (Experimental Procedures). Of the plated seeds, 73% of LD seeds and 75% of SD seeds germinated on the first day, with a mean germination rate per accession of 85% (Figure S1, Table S3). To account for differences in hypocotyl length that were due to delayed germination, we restricted our analysis to seedlings that germinated on the first day and excluded eight accessions with variable germination from further analysis.

**FIGURE 1:**
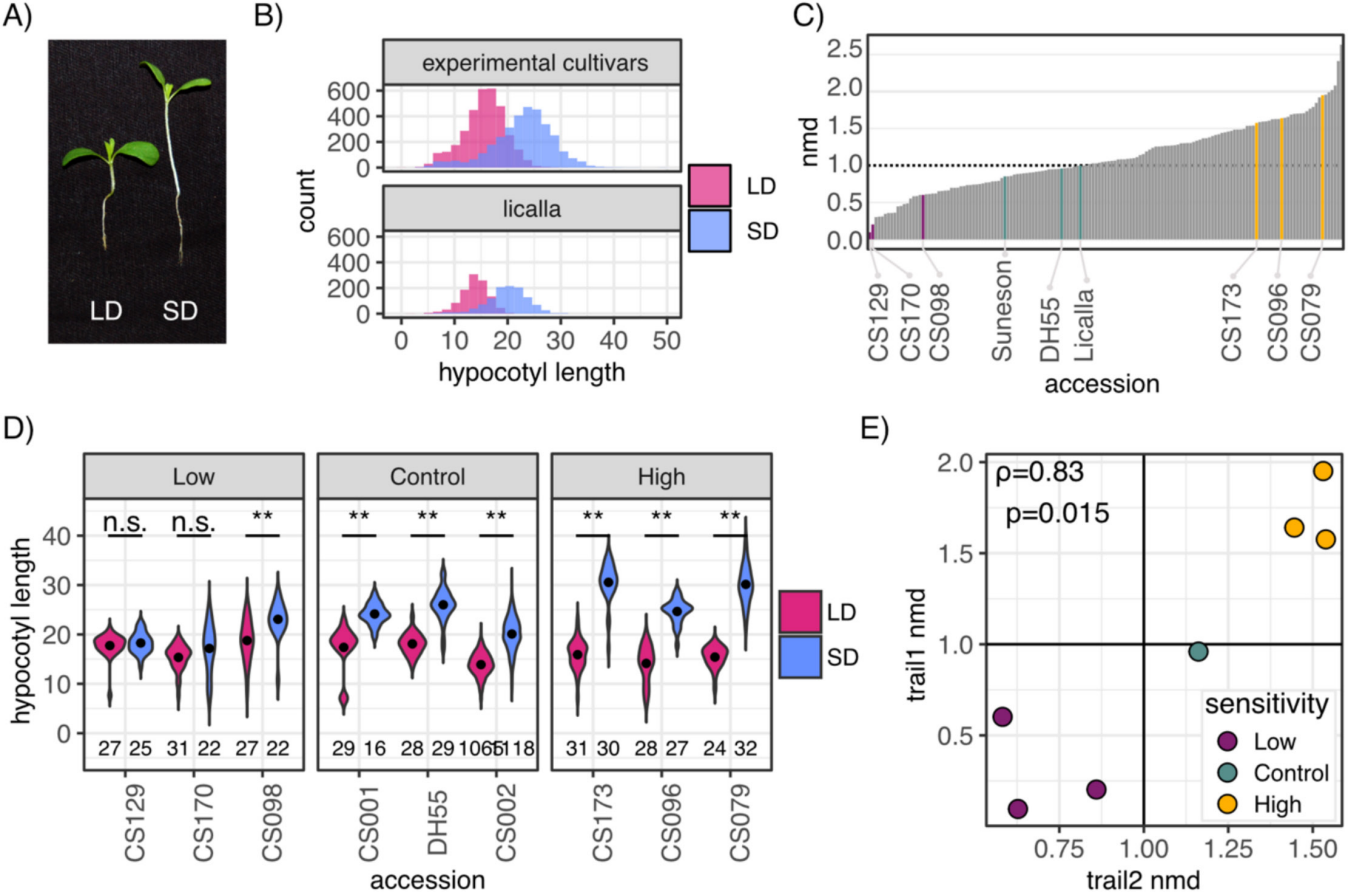
Quantification of the hypocotyl response to day length in 161 accessions of *Camelina sativa* . (A) Representative image of *C. sativa* seedlings (Licalla accession) grown under long‐day (LD) or short‐day (SD) conditions. (B) Distribution of hypocotyl lengths from 4‐day‐old seeds germinated on Day 1 for all experimental accessions and Licalla. LD treatment (magenta) yields shorter hypocotyls than SD (blue) treatment. (C) Normalized mean difference (NMD) for all accessions corrected by batch. Accessions with low hypocotyl response to day length (CS170, CS129, and CS098) are marked in purple, accessions with high response to photoperiod (CS173, CS096, and CS079) are marked in gold, and control accessions Licalla (CS002), DH55, and Suneson (CS001) are marked in green. All accessions show some degree of responsiveness to day length. (D) LD and SD hypocotyl lengths of selected accessions in both SD and LD treatments, X‐axis is ordered by increasing NMD (two‐sided *t*‐test, *: *p* ≤ 0.01, **: *p* ≤ 0.001, ***: *p* ≤ 0.0001). Two of the three accessions with low hypocotyl response to day length did not show significant differences in mean hypocotyl length between photoperiod treatments. *p*‐values: CS129 = 0.81, CS170 = 0.23, CS098 = 6.24 × 10–4, CS001 = 4.84 × 10–8,DH55 = 2.24 × 10–14, Licalla < 1.1 × 10–29, CS173 = 9.37 × 10–22, CS096 = 1.88 × 10–16, CS079 = 2.81 × 10–23. (E) Correlation of NMD values between the two trials of hypocotyl assays shows that the exact rank order is not preserved, but the differences between accessions with low and high hypocotyl response to day length remain. Trials show significant positive correlation with each other (Spearman rank correlation, *ρ* = 0.82, *p*‐value = 0.0068).

As expected, LD‐grown seedlings had generally shorter hypocotyls than SD‐grown seedlings (Figure 1A,B). To quantify differences in the hypocotyl response to day length among accessions, we calculated the mean difference (MD) in hypocotyl length for each accession between SD and LD conditions and adjusted for experimental batch effects (Experimental Procedures, Figure S2). To correct for batch effects, we included seedlings of the reference accession Licalla on all plates and calculated a normalized mean difference (NMD) in hypocotyl length by dividing each tested accession's MD value by the MD value of the respective Licalla seedlings (Experimental Procedures). In short, an accession fully lacking a hypocotyl response to day length would show an NMD of zero, while Licalla would show an NMD of 1.

*C. sativa* accessions varied greatly in NMD, with the well‐known accessions DH55 (Kagale et al. 2014) and Suneson (CS001, Li et al. 2021) showing lower NMDs than Licalla. Overall, 83 accessions showed NMD values greater than Licalla, while 67 showed lower NMD values. Most accessions showed significant differences in hypocotyl length between LD and SD (129 out of 147 testable accessions), including Licalla, DH55, and Suneson (Figure S3). Next, we selected six accessions with either low or high response to day length in hypocotyl length for testing adult traits, taking into account the effects size of their respective hypocotyl response and its significance. Because NMD is sensitive to germination effects and seedling health, we manually screened images of candidate accessions for high and consistent germination rates and robust seedling growth in both LD and SD conditions (Figure S4). Based on these criteria, we selected three accessions with a high response in hypocotyl length (CS173, CS096, and CS079) and three accessions (CS170, CS129, and CS098) with a low response (Figure 1D), in addition to the control accessions DH55 and Licalla.

While the selected accessions were grown to adulthood, we conducted a validation experiment that tested the hypocotyl response to day length of these accessions in a single experimental batch with twice the number of seedlings per test condition. The majority of seeds germinated on the first day, allowing us to focus our analysis on Day 1 seedlings (Figure S5A). In this assay, all three accessions with low hypocotyl response to day length showed significant differences between LD and SD conditions (Figure S5, CS129: *p*‐value = 3.63 × 10^−5^, CS170: *p*‐value = 1.79 × 10^−12^, CS098: *p*‐value = 1.27 × 10^−6^). This is likely due to the increased power in the validation experiment. Consistent with the previous trial, both the controls and the accessions with high hypocotyl response to day length showed significant differences between LD and SD. NMD measurements between the two trials were significantly correlated (Figure 1E, Spearman's *ρ* = 0.82, *p*‐value = 0.0068). Although the exact rank order of the hypocotyl responses to day length was not preserved between the two trials, the overall separation of accessions with high and low hypocotyl response was validated.

**FIGURE 2:**
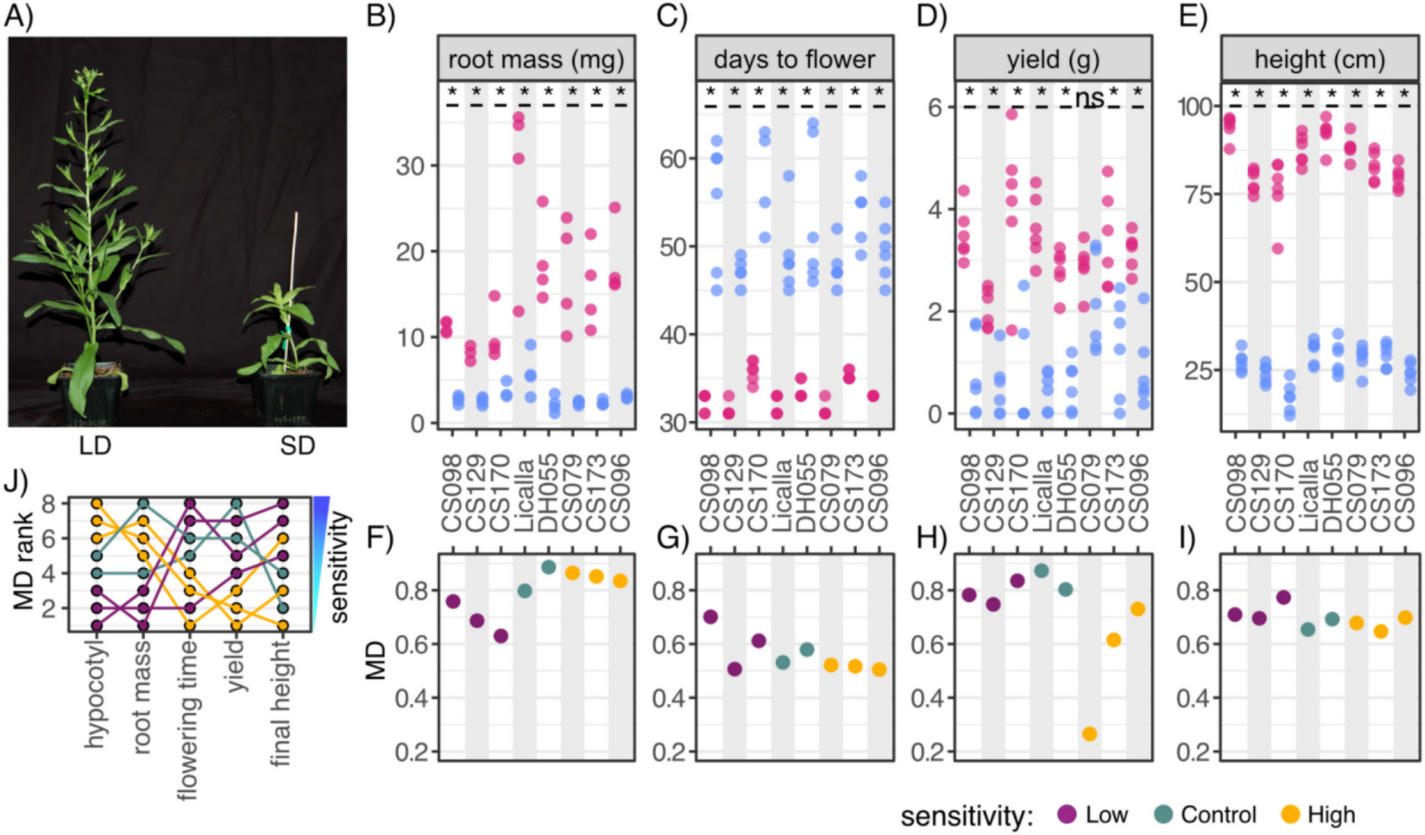
Rank order of sensitivity to day length is not maintained across traits. (A) Representative image of 5‐week‐old soil‐grown Camelina plants in LD (left) and SD (right) conditions. (B–E) Pink points indicate LD treatment, and blue points indicate SD treatment. X‐axis shows accessions ordered left to right by increasing hypocotyl length NMD. Y‐axis shows the measured adult trait listed at the top of the graph. (B) Root mass measured in 20‐day‐old plants with significant differences between SD and LD conditions labeled (two‐sided *t*‐test, * < *p*‐value 0.05). (C) All accessions showed significant differences in the number of days to flowering between SD and LD conditions (top, two‐sided *t*‐test, * < *p*‐value 0.05). (D) All accessions except CS079 showed significant differences in yield (total seed weight in g) between SD and LD conditions (two‐sided *t*‐test, * < *p*‐value 0.05). (E) All accessions showed significant differences in the height of the central stalk (cm) at 41 days, between SD and LD conditions (two‐sided *t*‐test, * < *p*‐value 0.05). (F–I) X‐axis shows accessions ordered left to right by increasing hypocotyl length NMD. Purple points indicate accessions with low photoperiod sensitivity in hypocotyls, yellow points indicate accessions with high photoperiod sensitivity in hypocotyls, and green points indicate control accessions. Y‐axis shows the corresponding MD value for each accession for corresponding trait in the graph above. (J) Ranked photoperiod response for the eight accessions across adult traits using MD for each trait.

We next asked whether these differences were maintained in adult traits. Height, root mass, flowering time, and seed yield are important agronomic traits for breeding and crop development and were quantified in adult plants. We grew 10 plants of each selected accession under LD and SD conditions. Four plants from each accession were used for gene expression measurements and root mass measurements at Day 20 (Experimental Procedures). The remaining six plants of each accession were grown for 65 days before drying for seed harvesting (Experimental Procedures). Contrary to our observations in the hypocotyl assay, the central stalk of soil‐grown plants in SD conditions were shorter in height than plants in LD conditions, consistent with reduced vegetative growth (Figure 2A). Considering the MD in adult plant height, the initial separation based on hypocotyl response to day length was lost as early as in the third week of plant growth and remained so until the end of the trial (Week 6, Figure S6). Neither the rank order nor the categories of accessions with high and low hypocotyl response to day length were preserved.

Root mass at Day 20, days to flowering and seed yield were also quantified for each accession. For both root mass and days to flowering, all accessions showed significant differences between LD and SD conditions (Figure 2B–E). Similarly, the total seed weight for each accession significantly increased in LD conditions, except for the CS079 accession for which there was no difference (Figure 2D). This observation is notable because this accession showed a high response to day length in the seedling trait hypocotyl length, but it is the least day length‐sensitive accession for the adult plant trait total seed weight. A plant's seed yield is most readily measured as total seed weight; however, seed weight divided by the total number of seeds produced (individual seed weight) is also highly informative for breeders. Here, we approximated this measure by weighing 100 seeds for each tested accession (individual seed weight). The average individual seed weight and estimated number of seeds were significantly correlated in the controls and the accessions with low hypocotyl response to day length, indicating that these accessions achieve higher total seed weight in LD by increasing both the total number of seeds and the individual seed weight (Figure S7A). The accessions with high hypocotyl response to day length, however, failed to show strong correlation between total seed number and average individual seed weight. Thus, the higher total seed weight in these accessions in LD conditions was the result of additional seeds of similar size that were markedly smaller than those in the accessions with low hypocotyl response to day length.

For each measured trait, we calculated the MD of each accession (Figure 2F–I), normalized by the Licalla MD value. Neither rank order nor the observed separation of low and high day length‐responsive accessions was maintained between our hypocotyl assay and the adult traits (Figure 2J). Spearman rank correlations of trait MD values showed no significant correlations between any of the adult traits (Figure S8). Due to this lack of concordance in MD rank across traits, none of the accessions can be singularly classified as less photoperiod‐sensitive than the others. This result is consistent with photoperiod response being a complex trait in this crop.

**FIGURE 3:**
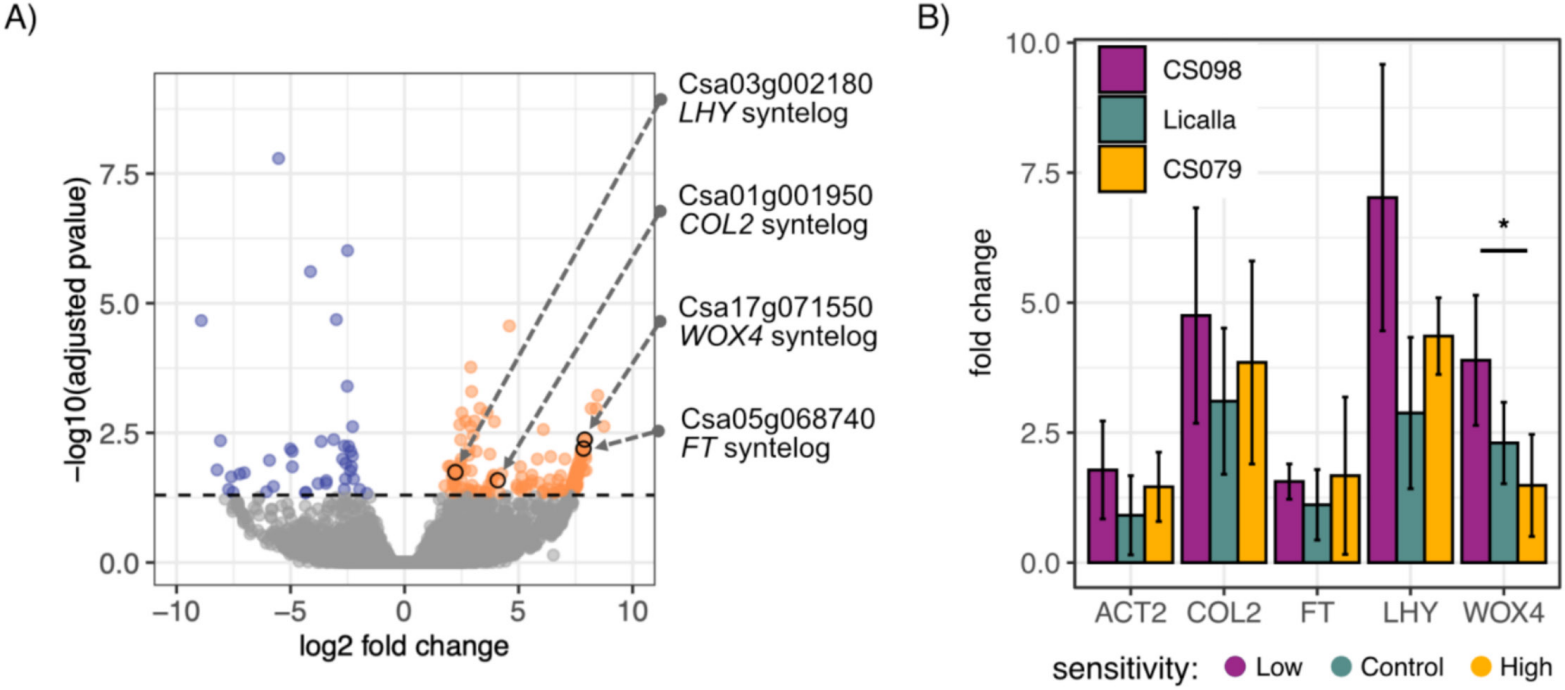
Expression of known photoperiod response genes and a developmental gene is higher in LD, but no expression differences are observed among accessions with low and high hypocotyl response to day length. (A) Expression differences in LD in 3‐week‐old Licalla leaves relative to SD. A total of 40,468 genes were detected with 151 genes upregulated and 67 downregulated. Syntelogs for potential photoperiod responsive genes *COL2*, *LHY*, and *FT* and the developmental gene *WOX4* are highlighted. (B) qPCR of the accession CS098 (purple, low hypocotyl response to day length), the control accession Licalla (green), and the accession CS079 (gold, high hypocotyl response to day length). Expression of the selected genes is higher in LD conditions. Expression of *WOX4* differs significantly between CS098 and CS079 accessions (two‐sided *t*‐test, *p* = 0.049, Bonferroni corrected); however, the remaining genes did not show significant expression differences. Columns show the mean fold change, and error bars show standard deviation.

Although much is known in *A. thaliana* about the photoperiod response and the genes that regulate it (Nagel and Kay 2012; Song et al. 2018), far less is known about how the *C. sativa* syntelogs behave and their utility as potential markers for phenotypic traits of interest. To address this knowledge gap, we performed bulk RNA‐sequencing on the aerial tissue of 3‐week‐old Licalla plants grown in either LD or SD conditions (Experimental Procedures). Of the detected 40,468 genes, 218 were found to be differentially expressed between LD and SD conditions. Specifically, 151 genes were upregulated, and 67 genes were downregulated in LD conditions, relative to SD (Figure 3A). Of these genes, 126 had known *Arabidopsis* orthologs, 98 were upregulated, and 28 were downregulated. Of the upregulated genes, four genes were of particular interest: *CONSTANS‐LIKE 2* (COL2), *FLOWERING LOCUS T* (*FT*)/*TWIN SISTER OF FT* (*TSF*), *LATE ELONGATED HYPOCOTYL* (*LHY*), and *WUSCHEL RELATED HOMEOBOX 4* (*WOX4*), syntelogs of *Arabidopsis* that are involved in photoperiod‐controlled developmental responses. *COL2* is a zinc finger protein with sequence similarity to the flowering gene *CONSTANS* and has been implicated in flowering time regulation in other plants (Ledger et al. 2001; Liu et al. 2021; Liang et al. 2023). *FT* is a florigen that, along with *TSF*, acts as a mobile signal to induce the vegetative to flowering transition (Song et al. 2015; Wang et al. 2020; Lee et al. 2023); both are syntelogs of the highly differentially expressed *C. sativa* gene Csa05g068740, which had nearly an eightfold change in expression between LD and SD. *LHY* is a core circadian clock gene that in *Arabidopsis* is involved in the regulation of several developmental processes including flowering time and the *FT* locus (Fujiwara et al. 2008 *;* Nagel and Kay 2012). While *WOX4* in *Arabidopsis* is primarily involved in cell division and vascular proliferation, several WOX transcription factors are involved in floral development (Costanzo et al. 2014). Other syntelogs of flowering time regulators, such as *EARLY FLOWERING 3* (ELF3), *GIGANTEA* (GI), and *CONSTANS* (CO), were not differentially expressed in our data (Figure S9, Nagel and Kay 2012). Although these selected examples had syntelogs in *Arabidopsis*, many other differentially expressed genes did not (*n* = 92) suggesting there are many more potential genes of interest to dissect.

Having identified genes upregulated in LD in Licalla plants, we next asked if expression differences of these genes could explain the differences observed between accessions in adult traits. We extracted RNA from aerial tissue of 20‐day‐old plants grown in either LD or SD, using one genotype with low hypocotyl response to day length (CS098), one with high hypocotyl response (CS079) and Licalla as a reference control. We performed qPCR using primers for *COL2*, *FT*, *LHY*, *WOX4*, *ACT2*, and *SEC3* (Table S2). *ACT2* was included as a control and SEC3 was used as the calibrator for calculating fold change (2^‐∆∆Cq^) relative to SD treatment (Data S3, Chao et al. 2019). All selected genes were upregulated in LD conditions. The only significant accession differences observed were in *WOX4* between the accessions with high or low hypocotyl response to day length (Figure 3B). We noted that in particular for *FT* and *WOX4*, the magnitude of expression differences between the RNA‐sequencing results and the PCR data was large (230‐fold vs. twofold). We suspect that this difference is in part due to the difference in the time of tissue collection (RNA‐seq ZT4; qPCR, ZT8). This discrepancy might also reflect that factors like FT are generally lowly expressed and localized to a few specialized cells (Takagi et al. in press). Nevertheless, the expression patterns of the selected genes were insufficient to tease apart the observed phenotypic differences among these accessions. Finding genes whose expression patterns better explain the phenotypic variation observed in Camelina photoperiod response will be necessary for future studies.

## 3 Discussion

Here, we quantified differences in the hypocotyl response to day length in a panel of 161 *C. sativa* accessions, observing a wide spectrum of variation in this seedling trait. Several accessions showed little difference in hypocotyl length between SD and LD conditions, appearing nearly unresponsive to day length in this early trait. Accessions were categorized as having either low or high hypocotyl response to day length, and three accessions at both ends of the phenotypic spectrum were selected for testing photoperiod‐associated adult traits. None of the selected accessions were found to be day neutral at the adult stage. We observed significant differences between LD and SD treatments in the adult traits of root mass, height, flowering time, and seed yield. However, there was no meaningful correlation between response to day length in the seedling trait hypocotyl length and photoperiod sensitivity in the measured adult traits. In fact, the rank order of photoperiod sensitivity differed across all the measured adult traits, suggesting trait‐specific responses associated with day length in the tested accession, while seasonal time measurement mechanisms were intact.

Our results suggest that day neutrality may not be present among existing *C. sativa* germplasms. If so, breeders cannot rely on introgression of day neutrality from existing accessions for the development of high‐yielding, day‐neutral *C. sativa* . Rather, genetic engineering of photoperiod measurement mechanisms will be required to generate such lines. In order to facilitate the identification of possible engineering targets, we conducted RNA‐seq with the reference accession Licalla. Of the 151 significantly upregulated genes, we selected four *Arabidopsis* syntelogs involved in photoperiod response, *COL2, FT*, *LHY*, and *WOX4*, for expression studies in low and high photoperiod‐sensitive accessions. However, none of the genes showed accession‐specific differences in expression. Other known *Arabidopsis* syntelogs, *CO*, *ELF3*, and *GI*, were detected in our data set but were not found to be differentially expressed between LD and SD; however, these genes are known to peak in the evening, whereas our data were collected in the morning (ZT4).

While hypocotyl assays provide a high‐throughput approximation for the function of the genetic networks involved in the photoperiod response, the results can be confounded by the effects of photomorphogenesis unrelated to photoperiod. Here, we controlled for the effects of total light received by increasing the light intensity in SD conditions (Experimental Procedures). Nevertheless, the expression of critical genes like the *PHYTOCHROME INTERACTING FACTORS PIF3* and *PIF4* and *ELONGATED HYPOCOTYL5* (*HY5*) could differ between SD and LD conditions (Krahmer and Fankhauser 2024). Thus far, however, *C. sativa* syntelogs for *PIF4* and *HY5* have not been identified, and the two *PIF3* syntelogs showed no difference in expression in our RNA‐seq data. Similarly, the addition of sucrose to the growth medium affects hypocotyl length and may confound conclusions about the response to day length. However, these sucrose effects are strongest in dark growth conditions and depend on the concentration of sucrose (Alexandre et al. 2024; Kircher and Schopfer 2012; Su et al. 2024; Zhang et al. 2010). Future experiments will have to determine to what extent the lessons learned in *A. thaliana* will apply to the photoperiod response in the hexaploid *C. sativa* . This study can only serve as starting point to explore the underpinnings of photoperiod sensitivity in this crop.

The molecular basis of day neutrality in other crops is complex (Lin et al. 2021; Wang et al. 2023). Studies in rice, tomato barley, and canola, among other crops, have uncovered some of the genes, and candidate loci involved in reducing photoperiod response and increasing yields (Turner et al. 2005; Comadran et al. 2012; Wang et al. 2016; Soyk et al. 2017; Wei et al. 2017; Liu et al. 2018; Zhang et al. 2018; Lu et al. 2019 *;* Song et al. 2020; Zong et al. 2021). In barley, these studies have yielded a complex picture with different alleles of *TERMINAL FLOWER1*/*CENTRORADIALIS* conferring an advantage under different environmental conditions (Comadran et al. 2012). In spring‐sown barley, a pseudo‐response regulator *Ppd‐H1* variant delays flowering specifically in long days, illustrating that variation in diverse genes associated with clock function and photoreception can confer a weaker photoperiod response (Turner et al. 2005). In canola, the world's second most important oilseed crop, several dozen loci contribute to variation in flowering time among cultivars, consistent with the crops complex allotetraploid nature (Schiessl 2020; Song et al. 2020). In tomato, domesticated day‐neutral lines have been found to have altered circadian rhythms that appear to confer fitness under LD conditions (Müller et al. 2015) In several crops, including tomato and rice, variation in regulatory DNA and changes in promoter enhancer interaction are implicated in the acquisition of day neutrality (Takahashi et al. 2009; Zhang et al. 2018). An attenuated photoperiod response is often associated with the loss for the vernalization requirement, the need for a “winter” period before flowering (Malik et al. 2018).

Without the benefit of *C. sativa* varieties with stark and consistent differences in photoperiod sensitivity, engineering this trait will be a formidable challenge. A first step toward disentangling the genetic underpinnings of photoperiod sensitivity in *C. sativa* would be detailed expression studies across development and tissues to shed light on genes that consistently show photoperiod‐sensitivity. Our simple expression experiment discovered 92 differentially expressed genes without *A. thaliana* syntelogs, providing a set of potentially interesting candidate genes for future investigations. Additionally, it would be useful to identify traits or sets of traits that are most predictive of day neutrality to facilitate the engineering and breeding of *C. sativa* varieties that combine day‐neutrality and high seed yields with the crop's other favorable agronomic properties.

## 4 Experimental Procedures

### 4.1 Accessions/Plant Materials and Growth Conditions/Camelina Cultivation

*C. sativa* stocks consisted of 160 accessions generously provided by Jennifer Lachowiec from Montana State University as well as DH55 from Agriculture Agri Food Canada (Kagale et al. 2014; Li et al. 2021). All accessions were seeded in soil (Sunshine Mix #4) and grown in one of two photoperiod conditions, LD (16‐h light, 8‐h dark; 250 μmol m^−2^ s^−1^; R:FR ratio = 1) or SD (8‐h light, 16‐h dark; 500 μmol m^−2^ s^−1^; R:FR ratio = 1) at 22°C. Valoya BX LEDs lights were used. For seed collection, plants were grown for ~9 weeks before water supply was slowly reduced to dry plants for harvesting. Seeds from plants grown under SD and LD conditions were combined into one seed stock per accession, which was used for all subsequent experiments. Seeds were stored in coin envelopes under open air conditions or in closed plastic containers containing desiccants (DRIERITE anhydrous calcium sulfate).

### 4.2 Hypocotyl Elongation Assay

To characterize potential day length‐dependent hypocotyl elongation differences, 161 *C. sativa* accessions were assayed under SD and LD conditions. Seeds were sown on clear square grid plates (Genesee Cat# 26‐275) containing Murashige and Skoog (MS) (PhytoTechnology Laboratories) agar media (1x MS basal salts, 1x MS vitamin powder, 1% sucrose, 0.3% phytagel, 0.5 g/L MES hydrate). Seeds were sterilized by 10‐min exposure to 70% ethanol and 0.5% Tween 20 (Thermo Fisher Scientific) followed by 5‐min exposure to 95% ethanol while being shaken vigorously. Sterile seeds were suspended in 0.1% agarose and eight seeds were pipetted onto each experimental plate. After plating, seeds were placed in the dark at 4°C for ~24 h to synchronize germination. The experimental plates were then split between LD growth conditions (16‐h light, 8‐h dark; 100 μmol m^−2^ s^−1^) or SD growth conditions (8‐h light, 16‐h dark; 125 μmol m^−2^ s^−1^). For each accession, four plates (32 seeds) were run per photoperiod condition, for a total of 64 experimental seeds per accession. Each condition and accession pairing was simultaneously run with one *C. sativa* Licalla plate as a control.

Following germination synchronization, seed plates were grown for 4 days in Conviron TC26 growth chambers. During the growth period, each day plate locations within the growth chamber were rotated, and germinated seeds were marked. At the end of the growth period, plates were examined under a dissecting microscope, and the ends of the hypocotyls were marked prior to imaging.

### 4.3 Image Analysis

Image analysis was conducted using ImageJ (1.53, Schneider et al. 2012). Using a graphics tablet with stylus (Wacom Intuos3 PTZ1230), 10 measurements of the plate grid length were taken and averaged to set the pixel to millimeter scale. To measure the hypocotyl length, each hypocotyl was manually traced from end to end using the marks that were made under the microscope as guides. Contaminated seedlings were excluded.

### 4.4 Hypocotyl Assay Data Analysis

Data collected from the hypocotyl assay was imported into R (3.6.0, R Core Team 2019) for analysis, and ggplot2 (3.4.4) was used to generate figures. Only seeds that germinated on the first day of the growth period and that had a nonzero hypocotyl length were included in this analysis. Mean and standard deviation were calculated for each accession, and data more than three standard deviations from the mean were labeled as outliers and excluded from further analysis. To test for significant differences in hypocotyl length response for each accession, we used a two‐sided *t*‐test on hypocotyl length measurements between LD and SD.

To compare the response between accessions, the difference in mean hypocotyl length between LD‐grown seedlings and SD‐grown seedlings was calculated for each accession and then divided by the LD hypocotyl length of the accession (${\mathrm{MD}}_{\text{accession}}$). To account for batch effects, we divided accession MD values by the corresponding MD value of Licalla for the corresponding batch thus calculating the NMD.

$${\mathrm{MD}}_{\text{accession}}=\frac{\underline{\mathrm{LD}}\;\text{accession}-\underline{\mathrm{SD}\;}\text{accession}}{\underline{\mathrm{LD}}\;\text{accession}}\;\mathrm{NMD}=\frac{{\mathrm{MD}}_{\text{accession}}}{{\mathrm{MD}}_{\text{licalla}.}}$$

### 4.5 Soil‐Grown Assay

Six accessions selected for their high or low response of hypocotyl length to day length were grown in soil to determine their adult phenotypes in SD and LD. Two accessions were grown as controls: *C. sativa* Licalla whose photoperiod response was already characterized, and DH55, which was used to generate the *C. sativa* reference genome.

For each accession, 10 plants were grown under LD conditions, and 10 were grown under SD conditions as outlined in *Growth Conditions*. When the plants reached 3 weeks of age, four‐LD‐grown and four‐SD‐grown plants per accession were selected at random to be harvested. The aerial tissue, including leaves and stems, was separated from the root tissue and flash frozen in liquid nitrogen. Collected tissue was stored at −80°C prior to RNA extraction. The root tissue was washed of soil and debris and then dried at 80°C for 24 h prior to weighing.

The remaining six plants per accession and photoperiod condition were grown to adulthood. Weekly height measurements were taken from soil level to the top of the central stalk with a meter stick from Weeks 3 to 6. Each day, plants were checked, and the date of emergence of the first flower was recorded for each plant. After approximately 65 days, watering frequency was gradually reduced to allow the plants to dry for harvesting. Seeds were harvested and stored as in previous experiments; however, the seeds of each plant were stored individually and weighed. Additionally, to obtain an average mass per seed, 100 seeds from each plant were weighed.

### 4.6 RNA Extraction

Plant aerial tissue was stored in −80°C. For RNA extraction, tissue was ground with liquid nitrogen using a mortar and pestle and suspended in 10 mL of QIAzol reagent (Qiagen). This suspension was separated via centrifugation (10 min at 4000 g, 4°C) and 5 mL of the supernatant was mixed with 1‐mL chloroform. This mixture was centrifuged (15 min at 4000 g, 4°C), and the resulting aqueous phase was transferred to a new tube and incubated for 15 min at room temperature with 2.5‐mL high salt buffer (0.8‐M sodium citrate, 1.2‐M NaCl) and 2.5‐mL isopropanol to precipitate RNA. The precipitate mixture was centrifuged (30 min at 4000 g, 4°C), and the resulting pellet was washed with 10 mL of cold 70% ethanol prior to resuspension in 200 μL of RNase free water.

### 4.7 RNA‐Seq

RNA‐sequencing libraries were prepared using extracted RNA and Illumina Stranded mRNA Prep kit. Reference accession Licalla was grown to 3‐week‐old plants, and three samples from aerial tissue were prepared per photoperiod treatment at Zeitgeber Time 4 (ZT4, *n* = 6). Sequencing was performed on NextSeq2000. Reads were trimmed using Trim Galore (0.6.10, Kreuger n.d.) default settings. Alignment of trimmed reads to the *C. sativa* genome (Kagale et al. 2014) was done using STAR (2.7.11.b, Dobin et al. 2013) default settings, and counts were quantified using htseq‐counts (2.0.3, Putri et al. 2022) using specifications “‐m union ‐r pos ‐i gene_name ‐a 10 –stranded = no.” Count data were downloaded into R (3.6.0, R Core Team 2019), and differential expression analysis was conducted using DESeq2 (3.19, Love et al. 2014)

### 4.8 qPCR

Four samples were collected per accession (CS098, Licalla, and CS079) and condition (LD and SD) for qPCR (*N* = 18). Aerial tissue was collected from 3‐week‐old plants at ZT8 First strand synthesis was done using SuperScript IV first strand synthesis Kit with ezDNase and RNaseH treatment (Invitrogen: 11766050), and cDNA was purified using the Zymo DNA Clean and concentrator kit. RT‐qPCR was performed on a CFX Connect Real‐Time System (Bio‐Rad) using 2x KAPA HiFi HotStart ReadyMix, 0.4X SYBR, and 100‐mM primers. For amplification, the following program was used: initial denaturation at 98°C for 30 s, followed by 40 cycles of 98°C for 20 s, 61°C for 30 s, and 72°C for 15 s. SEC3A was utilized as the calibrator gene for calculating sample ∆Cq values. For tested accessions, ∆∆Cq was calculated relative to LD, and fold change was calculated as 2^‐∆∆Cq^. Primers are listed in Table S2.

## Peer Review

The peer review history for this article is available in the Supporting Information for this article.

## Supporting information


**Data S1.** Peer review.

**Figure S1.** **Accessions show variation in both germination time and proportion of seeds germinated.** (A) Over 70% of total seedlings germinated across all accessions on Day 1 (72.77% LD and 74.67% SD). Of the remaining seeds 7.89% LD and 4.88% germinated on Day 2, 3.81% LD and 4.88% SD germinated on Day 3 and 1.98% LD and 2.13% SD germinated on Day 4. The remaining seeds 13.54% LD and 14.25% SD did not germinate before the end of this assay (marked with NA on the X‐axis). (B) The proportion of total seeds that germinated for each accession. The mean (red line) germination rate across all lines was 84.94%.
**Figure S2. Mean hypocotyl length was significantly correlated with Licalla hypocotyl length when stratified by batch.** (A) Mean hypocotyl length of the tested accessions in each experimental batch (Y‐axis) was significantly correlated with mean hypocotyl length of the corresponding Licalla seedlings in LD (X‐axis) (Pearson correlation: *r* = 0.74, *p*‐value = 0.008). (B) This result was also found for SD conditions (*r* = 0.67, *p*‐value = 0.022).
**Figure S3. The majority of accessions show significant differences between SD and LD hypocotyl lengths.** Mean hypocotyl lengths in LD and SD treatments for each accession were tested for significant differences (two‐sided *t*‐test). Y‐axis shows the ‐log10(*p*‐value) for each accession (X‐axis). Accessions along the X‐axis are ordered left to right by increasing NMD score. Red horizontal line denotes the significance threshold; −log(0.01). The vertical dotted line is the location of Licalla on the X‐axis. Accessions with low hypocotyl response to day length (CS170, CS129, and CS098) are marked in purple, accessions with high hypocotyl response to day length (CS173, CS096, and CS079) are marked in gold, and Licalla (CS002) DH55 are marked in green. Overall, 129 accessions showed significant differences in hypocotyl length between SD and LD treatments, and 17 accessions did not. Fifteen accessions did not have enough Day‐0 seedlings with a length greater than 0, for significance testing.
**Figure S4. Selecting healthy accessions for trial 2 experiments.** Representative images of two accessions with low NMD and no significant differences between LD and SD hypocotyl lengths. Accession CS129 (NMD = 0.096, *p*‐value = 0.81) was considered healthy and selected for retesting. Accession CS095 (NMD = 0.3, *p*‐value = 0.58) was not considered healthy and therefore not selected for validation.
**Figure S5: Germination rate and hypocotyl length of LD and SD plants for selected low hypocotyl‐responsive, high hypocotyl‐responsive, and control accessions.** (A) The percent of seeds germinated on Day 1 (Y‐axis) for each accession (X‐axis) was greater than 68% (*n* = 44) for most accessions. Only accession CS129 in the SD condition showed a germination rate of 39% (*n* = 25). (B) Hypocotyl length in mm (Y‐axis) and accession numbers (X‐axis) for SD (blue) and LD (magenta) conditions. In concordance with the initial trial, control accessions and accessions with high hypocotyl response to day length showed significant differences between SD and LD growth conditions (two‐sided *t*‐test *: *p* ≤ 0.05, **: *p* ≤ 0.01). Significant difference in hypocotyl length in SD vs LD. *p*‐values: CS129 = 3.63 × 10^−5^, CS170 = 1.79 × 10^−12^, CS098 = 1.27 × 10^−6^, DH55 = 1.74 × 10^−38^, CS002/Licalla = 6.61 × 10^−36^, CS173 = 8.1 × 10^−38^, CS096 = 1.77 × 10^−33^, CS079 = 6.27 × 10^−30^.
**Figure S6. Photoperiod sensitivity in adult plant height varies among accessions over time.** (A) Measured height (cm) of soil‐grown plants in either LD (magenta) of SD (blue) growth conditions from Week 3 to Week 6. (B) Corresponding normalized mean difference for accessions from Week 3 to Week 6 (bottom). Accessions with low hypocotyl response to day length (CS170, CS129, and CS098) are marked in purple, accessions with high hypocotyl response to day length (CS173, CS096, and CS079) are marked in gold, and Licalla (CS002) and DH55 are marked in green.
**Figure S7. Average individual seed weight and estimated seed number are least correlated in accessions with high hypocotyl response to day length.** (A) Average individual seed weight was calculated by weighing 100 seeds from each plant and dividing this weight by 100. The estimated number of seeds for each plant was calculated by dividing the yield weight by the average individual seed weight. Estimated seed number (X‐axis) and the average individual seed weight (Y‐axis) are highly correlated in the control accessions Licalla and DH55 (Spearman rank correlation, *ρ* = 0.79, *p*‐value = 6.85 × 10^−6^). Similarly, though to a lesser extent, this correlation was also observed in accessions with low hypocotyl response to day length (Spearman rank correlation, *ρ* = 0.51, *p*‐value = 0.005). Accessions with high hypocotyl response to day length did not show a significant correlation (*ρ* = 0.15, *p*‐value = 0.4). Accessions with low hypocotyl response to day length (CS170, CS129, and CS098) are marked in purple, high accessions with high hypocotyl response to day length (CS173, CS096, and CS079) are marked in gold, and Licalla (CS002) DH55 are marked in green. (B) The correlation between average individual seed weight and estimated seeds is lost in accession with high hypocotyl response to day length.
**Figure S8: Trait NMD is not significantly correlated across tested Camelina accessions.** NMD values for hypocotyl length (hypocotyl), 20‐day root mass (root), final height (measured at Day 41), flowering time, and seed yield (yield) were pairwise correlated using Spearman’s rank correlation. No pairwise correlation was found to be significant, demarcated with “X” in the corresponding pairwise comparison.
**Figure S9: Common flowering time regulators are not differentially expressed between LD and SD conditions.** Expression differences in LD in 3‐week‐old Licalla aerial tissue relative to SD. A total of 40,468 genes were detected with 151 upregulated (orange) and 67 downregulated (blue). Genes not differentially expressed are labeled in gray. Syntelogs known to regulate flowering time in *Arabidopsis* without differences in expression are highlighted as black circles. These syntelogs include (A) *EARLY FLOWERING 3* (ELF3) (B) *GIGANTEA* (GI), and (C) *CONSTANS* (CO).
**Table S1:** Name and source information for the 161 accessions included in this study.
**Table S2:** Primer sequences for qPCR.
**Table S3:** Germination day summary.

## Data Availability

Transcriptomic data can be found in the NIH short read archive (https://submit.ncbi.nlm.nih.gov/subs/sra/) under BioProject ID: PRJNA1086893.
