# Supplementary material for: Sensitivity to Photoperiod Is a Complex Trait in Camelina sativa
Source: Plant Direct. 2025 Apr 15;9(4):e70071. doi: 10.1002/pld3.70071 (PMC11999801; doi:10.1002/pld3.70071)
Supplement: Supplementary file 1 — Data S1. Peer review. [file PLD3-9-e70071-s002.pdf]

Plant Direct
Open Access

Title:

Sensitivity to photoperiod is a complex trait in *Camelina sativa*

Authors:

Bryan A Ramirez-Corona[1], Erin Seagren[1], Carissa Sherman[1], Takato Imaizumi[2], Christine Queitsch[1,3*], Josh Cuperus[1*]

Decision Letter Round 1:

December 5, 2024

Dr. Josh T Cuperus
University of Washington Department of Genome Sciences
Seattle 98195

RE: Sensitivity to photoperiod is a complex trait in Camelina sativa

Dear Dr. Cuperus:

Thank you for submitting to Plant Direct. All required reviews have been returned and we have now finished our evaluation of your manuscript. In light of the reviewers' and editor's comments, further revisions are needed before the paper can be accepted for publication in Plant Direct.

Please view the editors' and reviewers' comments below and use their suggestions as a guide while you work on your revision.

When uploading the revised version of this article, please be sure to include the following:

-A word document that contains your response to the reviewers. You should respond to each reviewer comment and note the changes made to the manuscript. If you do not agree with a reviewer's comment and choose not to make a suggested revision, please explain why. Please try to provide as complete an answer as possible to each reviewer's criticisms in the "response to reviewers" section.
-A tracked changes document
- A clean version of the latest version of the manuscript

To upload your revision, please click the link below.
https://plantdirect.msubmit.net/cgi-bin/main.plex?el=A6Lr5ld6A4pCd2I3A9ftdY7FI92n0bXY4hePFLZL8wZ

In order to provide as timely a service as possible, we ask that your revision is resubmitted within three

months after receipt of this request. If an extension is needed, please send a request, along with a brief explanation, to the editorial office at plantdirect@wiley.com .

Thank you very much for giving us an opportunity to review your work. I look forward to receiving the next version.

Sincerely,
Hongtao Ji

-- Editor comments:

Please address the reviewers' comments in an appropriate manner. Additionally, the references (Zhang et al., 2010; Su et al., 2024) proposed by Reviewer 2 were listed as follows after contacting Reviewer 2.

Zhang Y, Liu Z, Wang L, Zheng S, Xie J, Bi Y. Sucrose-induced hypocotyl elongation of Arabidopsis seedlings in darkness depends on the presence of gibberellins. J Plant Physiol. 2010 Sep 15;167(14):1130-6. doi: 10.1016/j.jplph.2010.03.007. Epub 2010 Apr 28. PMID: 20430474.

Su T, Liu H, Wu Y, Wang J, He F, Li H, Li S, Wang L, Li L, Cao J, Lu Q, Zhao X, Xiang H, Lin C, Lu S, Liu B, Kong F, Fang C. Soybean hypocotyl elongation is regulated by a MYB33-SWEET11/21-GA2ox8c module involving long-distance sucrose transport. Plant Biotechnol J. 2024 Oct;22(10):2859-2872. doi: 10.1111/pbi.14409. Epub 2024 Jun 11. PMID: 38861663.

-- Reviewer comments:
Reviewer #1:

The manuscript of Ramirez-Corona and coauthors describes the photoperiodic response of several Camelina sativa crop accessions under long and short days. The authors investigate the accession response using hypocotyl length as a first proxy. Quantifying in several ways and using Licalla accession as a photoperiodic response normalisation standard, they define several accessions as low, high and non-responsive. Then, they again studied those selected accessions' hypocotyl length and different traits, namely root biomass, flowering, final height, and yield. The correlation between different traits is not maintained, and the photoperiodic sensitivity ranking is not preserved. Finally, they tried to decipher through RNA-seq a molecular response to photoperiod in the control line Licalla, and they specifically quantified, using qPCR, some classical responder genes in the selected accessions.
The manuscript is well-written and easy to follow, with valuable data that, in principle, could be published in the Journal. However, the experimental approach's logic is missing and needs a better explanation. Classically, the photoperiodic response is limited to the transition phase study, and a plant is defined as a Long Day, Short Day or Neutral, only considering the time to flower under different light regimes. As such, using hypocotyl length as a proxy to shorten the time in a screening way of performing the study is not, in my opinion, a conceptually correct experimental approach. Hypocotyls are prone to pure photo-morphogenetic effects unrelated to photoperiod. Smartly enough, the authors try to

compensate for the plant growth impact by increasing the total amount of light the plants receive under short days (250 vs 500 µmol m-2 sec-1). However, I remain concerned that light compensation is insufficient to avoid pure photo-morphogenetic effects. For instance, in a classic experiment performed by Jan Zeevaart about flowering and photoperiod, to avoid light intensity effects, he even cut the Perilla leaves in an exact proportion to match light incidence in different photoperiods. I expect that the expression of critical genes like PIF4 or HY5 is different in both conditions, shadowing core genes like the ones studied in later plant stages (FT, CO-like, for instance). Without a molecular or physiological study showing that hypocotyl length is a sound (and separate output from photomorphogenesis signalling) to describe the phenomenological effects observed, I remain sceptical about the proper use of the term photoperiodic response term in the manuscript. Indeed, their results point out that the independence of photoperiod for multiple unrelated physiological outputs is the norm in Camelina, making it impossible to define the accession sensitivity to SD/LD using such traits. Crosslinking between both pathways is evident in temperature perception and flowering time regulation regarding PIF4, ELF3 and PhyB. Still, they are effectors upstream of the photoperiodic response as many inputs like the circadian clock. Please extend the discussion about how photomorphogenesis and photoperiodic traits could be unrelated to Camelina and, if possible, reinforce molecularly by analysing PIF4 and PIF3 gene expression in your conditions to rule out photomorphogenesis's role in the initial plant establishment.
Regarding the molecular approaches, I'm not surprised about the low number of genes altered. Most proper photoperiodic players have a circadian regulation and, in many cases, a sharp peak that is impossible to detect without an appropriate analysis of several consecutive ZT points. In this regard, the authors found several orders of magnitude differences between RNA seq and q PCR, attributed to different timing in the samples. I agree with such an interpretation of the results, but experimental artefacts should not be discarded. Moreover, in most Brassicaceae and Solanaceae species, FT-like and orthologs of FT are extremely difficult to detect in RNA-seq, and even quantitative PCR gave misleading and problematic results in the past. The authors should be aware of the low number of cells expressing such genes and all concomitant dilution effects. On the other hand, the expression could remain unaltered, and the photoperiodic effect could still be feasible. For instance, many CO orthologs could not be differentially expressed, and their molecular activity differs at the protein level.
I don't understand the inclusion of WOX4 as a photoperiod-responsive gene. If WOX4 is expressed in aerial tissue with high expression values and not limited to stem cell organising centre in the SAM, as WUSHEL, the logical conclusion is that it is the ortholog of the Arabidopsis AtWOX4, a gene related to vascular cambium and leaf development. Then, the differences between LD and SD are probably due to the cambium size but unrelated to core photoperiodic responses. Please explain the inclusion of AtWOX4.
In summary, I agree with publishing negative results like the ones presented in the manuscript. I think it is crucial in science to do it more openly, reliably, and sincerely, and I congratulate the authors for doing so. The data presented are valuable per se, and I think they should be published. Still, the paper's conclusion can stress more the complexity of photoperiod studies and the difficulties the authors deal with.

Reviewer #2:

The study investigated the natural variation in photoperiod sensitivity across 161 accessions of the oilseed crop Camelina sativa. The researchers found that all tested accessions showed some degree of

photoperiod response, with a wide range of sensitivity levels. While some well-known accessions like DH55 and Suneson were less photoperiod sensitive than the reference accession Licalla, the majority of accessions were more sensitive. The photoperiod responses observed in seedling traits, such as hypocotyl length, did not correlate with responses in adult plant traits, suggesting the underlying mechanisms are complex. This paper has some significance in the study of photoperiodic response in Camelina sativa, and the language is relatively fluent, which helps guide the reader's thought process. However, I think there are still some flaws that need to be addressed.

The most important issue here revolves around the use of hypocotyl length to reflect the photoperiodic response. Figure 1 and Supplemental Figure 5 show that the hypocotyl lengths of three low photoperiod-sensitive accessions, CS129, CS170, and CS098, are almost identical under both long and short day conditions. However, I seem to observe a trend where the hypocotyls are slightly shorter under long-day conditions. I am curious whether, after removing the extreme data points greater than three standard deviations from the mean, the hypocotyl lengths under long and short day conditions would still be considered not significantly different.

Additionally, I noticed that the significance levels used to claim no significant difference in hypocotyl length under long and short days were set at 0.01 (*), 0.001 (**), and 0.0001 (***). In general, in biological statistics, significance levels are typically set at 0.05 (*) or 0.01 (**). It may not be necessary to use a significance level below 0.01, or perhaps just presenting the raw p-values would be more appropriate.

The reason I am particularly concerned with this issue is that, according to some studies (Zhang et al., 2010; Su et al., 2024), sugar can affect hypocotyl length. More sugar tends to shorten the hypocotyl, and since photosynthesis duration differs under long and short days, there may be more sugar transported to the hypocotyls under long days. This could result in slightly shorter hypocotyls under long days, even after excluding the effects of photoperiodic response and photomorphogenesis.

If, after removing the extreme values, there is still no significant difference, it may suggest that the regulation of hypocotyl length in Camelina sativa differs from other plants. Please discuss this phenomenon in the discussion section. On the other hand, if significant differences are found after removing the extreme values, I don't think the impact on the results would be substantial, as the difference is indeed very small. Please also address this phenomenon in the discussion section.

Another issue I am concerned about is that the RNA-seq only sampled one time point (ZT4), and based on the data from this time point, it concludes that there is no expression difference in some genes under long and short photoperiods. However, some genes, such as ELF3, have different rhythms under long and short photoperiods, and the timing of their expression peaks is also different. I suggest that a 24-hour sampling (every 4 hours) be conducted for some important photoperiodic response genes (ELF3, GI, CO) to determine whether their expression levels really do not show significant differences under long and short photoperiods in Camelina sativa.

The last question is, where is Figure 4 mentioned in line 231?

Decision Letter Round 2:

April 2, 2025

Dr. Josh T Cuperus
University of Washington Department of Genome Sciences
Seattle 98195

MSID: 2024-01661-TWR1
MS TITLE: Sensitivity to photoperiod is a complex trait in Camelina sativa

Dear Dr. Josh Cuperus:

I am pleased to inform you that your manuscript "Sensitivity to photoperiod is a complex trait in Camelina sativa" has been accepted for publication in Plant Direct.

Your article will appear online in the next available issue of Plant Direct. To ensure your article gets published as quickly as possible, please pay attention to the steps detailed below. We have found that most of the delays happen at this stage, especially at the payment stage, so please respond as quickly as possible when prompted.

License Agreement: Once your article has been accepted it will move to Production and undergo admin and file checking - you may receive an email with any queries we have at this stage. When all required items are received by the publisher and queries resolved, the corresponding author will receive an email from Wiley's Author Services system which will ask them to log on at https://authorservices.wiley.com/bauthor and will present them with the appropriate license for completion. Your article cannot be published until both the signed license agreement and payment of the article fee have been received.

Payment of the Open Access Article Publication Fee: All articles published in Plant Direct are fully open access: immediately and freely available to read, download and share. Plant Direct charges a publication fee to cover the publication costs. The corresponding author for this manuscript should have already received a quote from the payments team (cs-openaccess@wiley.com) with the estimated article publication fee; please email cs-openaccess@wiley.com if this has not been received. The corresponding author should log on to the Wiley Author Services site, where the publication fee can be paid by credit card or an invoice. Pro Forma can also be requested. Payment of the publication charge must be received before the article will be published online.

Proofs: You will have the opportunity to look over your paper once more when you receive the author proofs for your article. The proofs will be with you in approximately two weeks. Please note that, in addition to publishing reviewer comments, the author's responses to review comments will also be published alongside the final version of the paper. If you would not like the author's responses to be published, please contact the editorial office at plantdirect@wiley.com .

Promotion of your article: You can help your research get the attention it deserves! Check out Wiley's free Promotion Guide for best-practice recommendations for promoting your work at www.wileyauthors.com/eeo/guide. And learn more about Wiley Editing Services which offers professional video, design, and writing services to create shareable video abstracts, infographics, conference posters, lay summaries, and research news stories for your research at www.wileyauthors.com/eeo/promotion.

Thank you again for your contribution to Plant Direct. If you have any questions, feel free to contact the editorial office at plantdirect@wiley.com .

Sincerely,

Plant Direct
Open Access

Hongtao Ji

Gustavo MacIntosh

Editor, Plant Direct

-- Reviewer comments:
Reviewer #1:

The author's responses and modifications in the manuscript fulfilled all my concerns and requirements, and it is correct to publish the manuscript in the current form.

Reviewer #2:

This manuscript is now ready for publication.

Response to Reviewer:

Resubmission request received : 12/5/2024
Resubmission due : 03/05/2025

-- Editor comments:

Please address the reviewers' comments in an appropriate manner. Additionally, the references (Zhang et al., 2010; Su et al., 2024) proposed by Reviewer 2 were listed as follows after contacting Reviewer 2.

Zhang Y, Liu Z, Wang L, Zheng S, Xie J, Bi Y. Sucrose-induced hypocotyl elongation of Arabidopsis seedlings in darkness depends on the presence of gibberellins. J Plant Physiol. 2010 Sep 15;167(14):1130-6. doi: 10.1016/j.jplph.2010.03.007. Epub 2010 Apr 28. PMID: 20430474.

Su T, Liu H, Wu Y, Wang J, He F, Li H, Li S, Wang L, Li L, Cao J, Lu Q, Zhao X, Xiang H, Lin C, Lu S, Liu B, Kong F, Fang C. Soybean hypocotyl elongation is regulated by a MYB33-SWEET11/21-GA2ox8c module involving long-distance sucrose transport. Plant Biotechnol J. 2024 Oct;22(10):2859-2872. doi: 10.1111/pbi.14409. Epub 2024 Jun 11. PMID: 38861663.

RESPONSE:
We thank the editor and the reviewers for their helpful comments on our manuscript. Below we address each comment in detail.

Reviewer #1:

The manuscript of Ramirez-Corona and coauthors describes the photoperiodic response of several

Plant Direct
Open Access

Camelina sativa crop accessions under long and short days. The authors investigate the accession response using hypocotyl length as a first proxy. Quantifying in several ways and using Licalla accession as a photoperiodic response normalisation standard, they define several accessions as low, high and non-responsive. Then, they again studied those selected accessions' hypocotyl length and different traits, namely root biomass, flowering, final height, and yield. The correlation between different traits is not maintained, and the photoperiodic sensitivity ranking is not preserved. Finally, they tried to decipher through RNA-seq a molecular response to photoperiod in the control line Licalla, and they specifically quantified, using qPCR, some classical responder genes in the selected accessions.

1a) The manuscript is well-written and easy to follow, with valuable data that, in principle, could be published in the Journal. However, the experimental approach's logic is missing and needs a better explanation. Classically, the photoperiodic response is limited to the transition phase study, and a plant is defined as a Long Day, Short Day or Neutral, only considering the time to flower under different light regimes. 1b) As such, using hypocotyl length as a proxy to shorten the time in a screening way of performing the study is not, in my opinion, a conceptually correct experimental approach. 1c) Hypocotyls are prone to pure photo-morphogenetic effects unrelated to photoperiod. Smartly enough, the authors try to compensate for the plant growth impact by increasing the total amount of light the plants receive under short days (250 vs 500 µmol m-2 sec-1). However, I remain concerned that light compensation is insufficient to avoid pure photo-morphogenetic effects. For instance, in a classic experiment performed by Jan Zeevaart about flowering and photoperiod, to avoid light intensity effects, he even cut the Perilla leaves in an exact proportion to match light incidence in different photoperiods. I expect that the expression of critical genes like PIF4 or HY5 is different in both conditions, shadowing core genes like the ones studied in later plant stages (FT, CO-like, for instance). Without a molecular or physiological study showing that hypocotyl length is a sound (and separate output from photomorphogenesis signalling) to describe the phenomenological effects observed, I remain skeptical about the proper use of the term photoperiodic response term in the manuscript. Indeed, their results point out that the independence of photoperiod for multiple unrelated physiological outputs is the norm in Camelina, making it impossible to define the accession sensitivity to SD/LD using such traits. Crosslinking between both pathways is evident in temperature perception and flowering time regulation regarding PIF4, ELF3 and PhyB. Still, they are effectors upstream of the photoperiodic response as many inputs like the circadian clock. Please extend the discussion about how photomorphogenesis and photoperiodic traits could be unrelated to Camelina and, if possible, reinforce molecularly by analysing PIF4 and PIF3 gene expression in your conditions to rule out photomorphogenesis's role in the initial plant establishment.

RESPONSE:
1a, b) As the reviewer rightly notes, the photoperiod response has been historically defined by the time to flowering. We have clarified this usage in the revised manuscript.

We would like to note that the genetic networks governing differences in 'hypocotyl elongation in short and long days' and 'time to flowering' are tighly interlinked in camelina's close relative *Arabidopsis thaliana*. Mutants in numerous light-sensing and circadian genes affect both traits. In fact, a key gene affecting time to flowering in response to photoperiod is LATE ELONGATED HYPOCOTYL (Park et al., 2016). There have been several studies in *Arabidopsis*, including our own, which have used the assessment of hypocotyl length in a similar context (Huang et al., 2016; Kim et al., 2005; Mizoguchi et al., 2002; Ronald et al., 2024; Sellaro et al., 2012; Undurraga et al., 2012; Zagotta et al., 1996).

As the reviewer states, we kept the total amount of light experienced by seedlings and mature plants constant – the only variable in these experiments was day length. We further note that the domestication events that have led to day neutrality in common crops such as rice, barley and tomato affect the same circadian gene networks that are known to affect hypocotyl length differences between short and long days in *Arabidopsis* (Comadran et al., 2012; Goretti et al., 2017; Liu et al., 2018; Matsubara et al., 2012; Soyk et al., 2017; Takahashi et al., 2009; Turner et al., 2005; Xue et al., 2008; B. Zhang et al., 2019; S. Zhang et al., 2018). Therefore, we feel justified in screening hypocotyls as a high-throughput proxy to explore the 161 camelina accessions for possible differences in the function of the gene networks associated with the photoperiod response. **In the revised manuscript**, we acknowledge throughout that the photoperiod response has been historically defined by the 'time to flowering' and explain our reasoning for screening the hypocotyl response to photoperiod **(see lines 89-96 in the introduction, lines 114-126 in the results, lines 422-424 in the discussion)**. We have replaced references to the "photoperiod response of hypocotyls" with the term "hypocotyl response to day length" throughout the manuscript.

1c) We acknowledge the reviewer's concern that photomorphogenic effects may confound our results and therefore explored the expression of PIF4 and HY5 as they suggested. However, neither PIF4 nor HY5 have syntelogs in the existing camelina genome annotation. There are three syntelogs for PIF3, but only two were detected in our RNA-seq experiment. As shown in the figure below, neither of the two syntelogs (indicated as black open circles) showed significant expression differences between LD and SD treatment in 3-week-old Licalla aerial tissue. In this experiment, a total of 40,468 genes were detected with 151 upregulated (orange) and 67 down regulated (blue). Genes that were not found to be differentially expressed are labeled in gray.

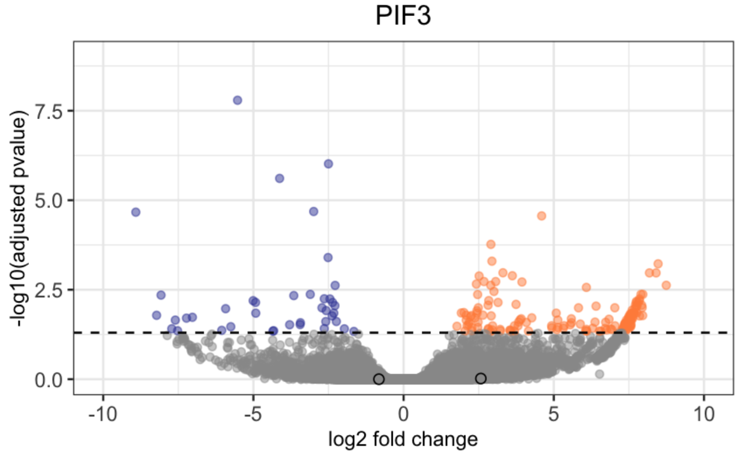

In light of this data, we find it unlikely that PIF3 is differentially regulated between SD and LD conditions among the tested accessions. In *Arabidopsis thaliana*, PIF3 is degraded in hypocotyls in the presence of light to promote photomorphogenesis (Dong et al., 2017). Therefore, it might require careful calibration to identify the right time point to detect and compare the expression of the PIF3 syntelogs between LD and SD conditions. Given these considerations, we think this additional effort is not justified. However, **in the revised manuscript**, we have expanded the discussion to include this potential source of confounding signal and a description of the data shown above (**see lines 426-430 in the discussion**).

2) Regarding the molecular approaches, I'm not surprised about the low number of genes altered. Most proper photoperiodic players have a circadian regulation and, in many cases, a sharp peak that is

impossible to detect without an appropriate analysis of several consecutive ZT points. In this regard, the authors found several orders of magnitude differences between RNA seq and q PCR, attributed to different timing in the samples. I agree with such an interpretation of the results, but experimental artefacts should not be discarded. Moreover, in most Brassicaceae and Solanaceae species, FT-like and orthologs of FT are extremely difficult to detect in RNA-seq, and even quantitative PCR gave misleading and problematic results in the past. The authors should be aware of the low number of cells expressing such genes and all concomitant dilution effects. On the other hand, the expression could remain unaltered, and the photoperiodic effect could still be feasible. For instance, many CO orthologs could not be differentially expressed, and their molecular activity differs at the protein level.

RESPONSE:
We agree with the reviewer and have added these thoughts to the results, specifically in regard to capturing FT expression (see **lines 365-366**). Please also see our very recent related paper (Takagi et al., 2025).

3) I don't understand the inclusion of WOX4 as a photoperiod-responsive gene. If WOX4 is expressed in aerial tissue with high expression values and not limited to stem cell organising centre in the SAM, as WUSHEL, the logical conclusion is that it is the ortholog of the Arabidopsis AtWOX4, a gene related to vascular cambium and leaf development. Then, the differences between LD and SD are probably due to the cambium size but unrelated to core photoperiodic responses. Please explain the inclusion of AtWOX4.

RESPONSE:
The reviewer is correct that WOX4 is not functioning in the photoperiod response. We examined expression of this gene because it was differentially expressed in our RNA-seq experiment and because of its known involvement in cambium development. As we report, there are profound differences in growth between SD plants and LD plants. As the reviewer notes, differences in WOX4 expression would be consistent with differences in cambium size between the smaller SD plants and the larger LD plants. Indeed, our qPCR results show that WOX4 is always upregulated in LD relative to SD in the three examined accessions. **In the revised manuscript**, we have clarified that WOX4 is not taking part in the photoperiod response but rather is a developmental gene (**see lines 99-100**).

4) In summary, I agree with publishing negative results like the ones presented in the manuscript. I think it is crucial in science to do it more openly, reliably, and sincerely, and I congratulate the authors for doing so. The data presented are valuable per se, and I think they should be published. Still, the paper's conclusion can stress more the complexity of photoperiod studies and the difficulties the authors deal with.

RESPONSE:
We thank the reviewer for their kind comments. We have expanded the discussion as requested (see **lines 422-436**).

Reviewer #2:

The study investigated the natural variation in photoperiod sensitivity across 161 accessions of the oilseed crop Camelina sativa. The researchers found that all tested accessions showed some degree of photoperiod response, with a wide range of sensitivity levels. While some well-known accessions like DH55 and Suneson were less photoperiod sensitive than the reference accession Licalla, the majority of accessions were more sensitive. The photoperiod responses observed in seedling traits, such as hypocotyl length, did not correlate with responses in adult plant traits, suggesting the underlying mechanisms are

complex. This paper has some significance in the study of photoperiodic response in Camelina sativa, and the language is relatively fluent, which helps guide the reader's thought process. However, I think there are still some flaws that need to be addressed.

5) The most important issue here revolves around the use of hypocotyl length to reflect the photoperiodic response. Figure 1 and Supplemental Figure 5 show that the hypocotyl lengths of three low photoperiod-sensitive accessions, CS129, CS170, and CS098, are almost identical under both long and short day conditions. However, I seem to observe a trend where the hypocotyls are slightly shorter under long-day conditions. I am curious whether, after removing the extreme data points greater than three standard deviations from the mean, the hypocotyl lengths under long and short day conditions would still be considered not significantly different.

RESPONSE:

We thank this reviewer for their comment regarding our hypocotyl data, particularly for the suggestion that outlier removal may reveal significant differences. In reviewing our data and while performing the suggested analysis, we realized an error in our analysis code for significance testing for accessions in the validation trial (*i.e.*, trial 2). In the validation trial, we erroneously performed significance testing before filtering for seedlings that germinated after day 1 and removing hypocotyls of length = 0. This error artificially inflated p-values in trial 2, resulting in two of the three accessions showing no significant differences in hypocotyl response between LD and SD treatments. After we corrected this error, all three accessions show significant differences. The remaining accessions from trial 2 (DH55, Licalla, CS173, CS096 and CS079) also showed an increase in significance.

In the figure below we have plotted the data for the accessions with low hypocotyl response to day length (CS129, CS170 and CS098). Hypocotyl length is plotted on the y-axis and accession numbers are on the x-axis. For transparency, we have overlaid the individual measurement points onto the violin plots and included the number of measurements below each plot. The data is separated by trial (rows) and by outlier filtering (columns). For outlier filtering, data points greater than 3 standard deviations from the mean where removed (right column). For trial 1 (Top row) outlier filtering removes only one data point from LD-treated CS129 and one data point form SD-treated CS098. Notably, this filtering step drastically affects the significance of accession CS098 (from p-value =0.011 to p-value=0.00031).

For trial 2 (bottom row), we first corrected the error in our original analysis code. On the left, are the p-values calculated with the correct filtering steps before significance testing. All three accessions now show significant differences in hypocotyl response. Outlier filtering (right column) further decreases our p-values. These values, however, do not change the calculated NMD rankings for these accessions, and do not change any of our previous conclusions. Please note that the six accessions we selected for additional testing were chosen on the basis of their NMD values relative to Licalla, LD vs SD hypocotyl p-values and general accession health. Thus, **despite implementing the suggested outlier filtering, the selected accessions represent the lowest and the highest measures of hypocotyl response to short and long days**.

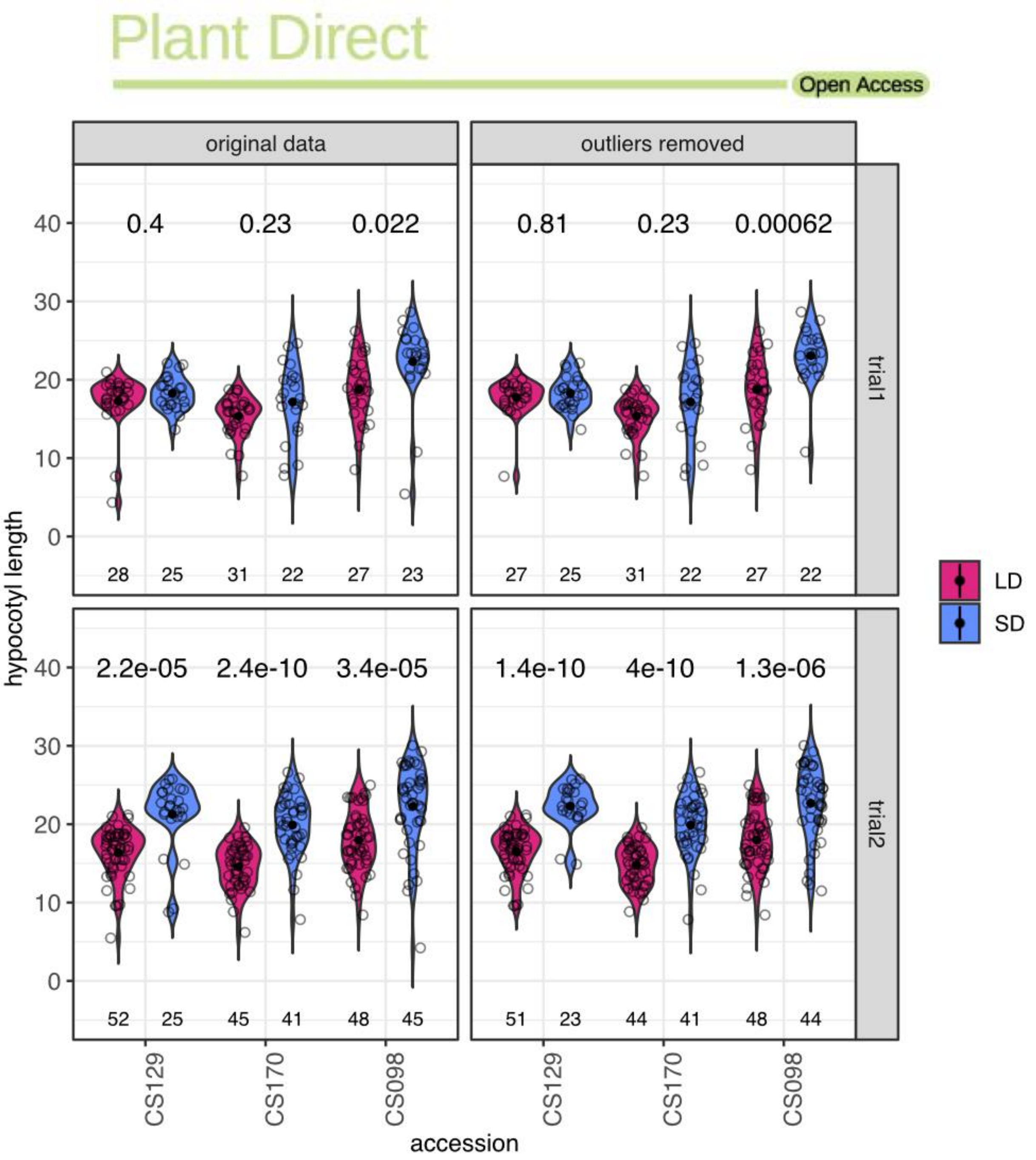

In the revised manuscript, we have incorporated outlier filtering in our hypocotyl analysis and updated figure 1, supplemental figure 2, supplemental figure 3 and supplemental figure 4 as well as our results and experimental procedures to reflect this change.

6a) Additionally, I noticed that the significance levels used to claim no significant difference in hypocotyl length under long and short days were set at 0.01 (*), 0.001 (**), and 0.0001 (***). In general, in biological statistics, significance levels are typically set at 0.05 (*) or 0.01 (**). It may not be necessary to use a significance level below 0.01, or perhaps just presenting the raw p-values would be more appropriate.

6b)The reason I am particularly concerned with this issue is that, according to some studies (Zhang et al., 2010; Su et al., 2024), sugar can affect hypocotyl length. More sugar tends to shorten the hypocotyl, and since photosynthesis duration differs under long and short days, there may be more sugar transported to

the hypocotyls under long days. This could result in slightly shorter hypocotyls under long days, even after excluding the effects of photoperiodic response and photomorphogenesis.
If, after removing the extreme values, there is still no significant difference, it may suggest that the regulation of hypocotyl length in Camelina sativa differs from other plants. Please discuss this phenomenon in the discussion section. On the other hand, if significant differences are found after removing the extreme values, I don't think the impact on the results would be substantial, as the difference is indeed very small. Please also address this phenomenon in the discussion section.

RESPONSE:

6a) We reasoned that a more stringent p-value might be necessary to confidently assign significance in hypocotyl response given the limited number of hypocotyl measurements per accession and the amount of variability observed in trial 1. In response to the prior comment and this one, we have applied outlier removal to our data and changed our threshold to a p-value of 0.05. Although this change affects the significance values of line CS098, the overall ranking of NMD values or any of the other conclusions are not affected. Additionally, the reviewer suggested the display of p-values in the figures; however, we found that this option crowded the figure panels, making them hard to read. Therefore, we continued to use the common asterisks annotations “0.05 (*)” or “0.01 (**)”. The p-values are now included in the figure legends.

6b) In *Arabidopsis*, sucrose in the growth media can inhibit or promote hypocotyl elongation under continuous light/dark conditions, but the effect has been described as relatively minor, dependent on sucrose concentration, and primarily affects skotomorphogenesis (Alexandre et al., 2023; Kircher & Schopfer, 2012; Y. Zhang et al., 2010). However, as mentioned, in soybean sucrose supplementation in LD does lead to shorter hypocotyls (Su et al., 2024). In the latter study, the sucrose concentration was 60mM; our sucrose concentration was 30mM. As the reviewer mentions, the effect is likely to be minor but we cannot exclude the possibility of this confounding signal. To address this concern the discussion has been updated to mention the potential effect of sucrose (**lines 431-434**).

7)Another issue I am concerned about is that the RNA-seq only sampled one time point (ZT4), and based on the data from this time point, it concludes that there is no expression difference in some genes under long and short photoperiods. However, some genes, such as ELF3, have different rhythms under long and short photoperiods, and the timing of their expression peaks is also different. I suggest that a 24-hour sampling (every 4 hours) be conducted for some important photoperiodic response genes (ELF3, GI, CO) to determine whether their expression levels really do not show significant differences under long and short photoperiods in Camelina sativa.

RESPONSE:

We agree that an RNA-seq time course would be nice to have. However, we are wary of conducting these laborious and expensive experiments because neither our current data nor published studies in other crops directly implicate the altered expression of these key genes in the acquisition of day neutrality. Furthermore, altered expression of these key genes should affect both seedling and adult traits – which is not what we observe in our study. In short, we are not convinced that these experiments would yield information that would fundamentally alter the message of this paper.

8) The last question is, where is Figure 4 mentioned in line 231?

RESPONSE:

We thank the reviewer for their attention to detail and for finding yet another error in our original manuscript. “Figure 4b” has been corrected to “Figure 3b” in the text.

Cited papers:

- Alexandre, C. M., Bubb, K. L., Schultz, K. M., Lempe, J., Cuperus, J. T., & Queitsch, C. (2023). LTP2 hypomorphs show genotype-by-environment interaction in early seedling traits in Arabidopsis thaliana. *The New Phytologist*. https://doi.org/10.1111/nph.19334
- Comadran, J., Kilian, B., Russell, J., Ramsay, L., Stein, N., Ganal, M., Shaw, P., Bayer, M., Thomas, W., Marshall, D., Hedley, P., Tondelli, A., Pecchioni, N., Francia, E., Korzun, V., Walther, A., & Waugh, R. (2012). Natural variation in a homolog of Antirrhinum CENTRORADIALIS contributed to spring growth habit and environmental adaptation in cultivated barley. *Nature Genetics*, *44*(12), 1388–1392.
- Dong, J., Ni, W., Yu, R., Deng, X. W., Chen, H., & Wei, N. (2017). Light-dependent degradation of PIF3 by SCFEBF1/2 promotes a photomorphogenic response in Arabidopsis. *Current Biology: CB*, *27*(16), 2420-2430.e6.
- Goretti, D., Martignago, D., Landini, M., Brambilla, V., Gómez-Ariza, J., Gnesutta, N., Galbiati, F., Collani, S., Takagi, H., Terauchi, R., Mantovani, R., & Fornara, F. (2017). Transcriptional and post-transcriptional mechanisms limit heading date 1 (Hd1) function to adapt rice to high latitudes. *PLoS Genetics*, *13*(1), e1006530.
- Huang, H., Yoo, C. Y., Bindbeutel, R., Goldsworthy, J., Tielking, A., Alvarez, S., Naldrett, M. J., Evans, B. S., Chen, M., & Nusinow, D. A. (2016). PCH1 integrates circadian and light-signaling pathways to control photoperiod-responsive growth in Arabidopsis. *ELife*, *5*, e13292.
- Kim, W.-Y., Hicks, K. A., & Somers, D. E. (2005). Independent roles for EARLY FLOWERING 3 and ZEITLUPE in the control of circadian timing, hypocotyl length, and flowering time. *Plant Physiology*, *139*(3), 1557–1569.
- Kircher, S., & Schopfer, P. (2012). Photosynthetic sucrose acts as cotyledon-derived long-distance signal to control root growth during early seedling development in Arabidopsis. *Proceedings of the National Academy of Sciences of the United States of America*, *109*(28), 11217–11221.
- Liu, H., Li, Q., & Xing, Y. (2018). Genes contributing to domestication of rice seed traits and its global expansion. *Genes*, *9*(10), 489.
- Matsubara, K., Ogiso-Tanaka, E., Hori, K., Ebana, K., Ando, T., & Yano, M. (2012). Natural variation in Hd17, a homolog of Arabidopsis ELF3 that is involved in rice photoperiodic flowering. *Plant & Cell Physiology*, *53*(4), 709–716.
- Mizoguchi, T., Wheatley, K., Hanzawa, Y., Wright, L., Mizoguchi, M., Song, H. R., Carré, I. A., & Coupland, G. (2002). LHY and CCA1 are partially redundant genes required to maintain circadian rhythms in Arabidopsis. *Developmental Cell*, *2*(5), 629–641.
- Park, M.-J., Kwon, Y.-J., Gil, K.-E., & Park, C.-M. (2016). LATE ELONGATED HYPOCOTYL regulates photoperiodic flowering via the circadian clock in Arabidopsis. *BMC Plant Biology*, *16*(1). https://doi.org/10.1186/s12870-016-0810-8
- Ronald, J., Lock, S. C. L., Claydon, W., Zhu, Z., McCarthy, K., Pendlington, E., Redmond, E. J., Vong, G. Y. W., Stanislas, S. P., Davis, S. J., Quint, M., & Ezer, D. (2024). Hypocotyl development in Arabidopsis and other Brassicaceae displays evidence of photoperiodic memory. In *bioRxiv*. https://doi.org/10.1101/2024.05.13.593876
- Sellaro, R., Pacín, M., & Casal, J. J. (2012). Diurnal dependence of growth responses to shade in Arabidopsis: role of hormone, clock, and light signaling. *Molecular Plant*, *5*(3), 619–628.
- Soyk, S., Müller, N. A., Park, S. J., Schmalenbach, I., Jiang, K., Hayama, R., Zhang, L., Van Eck, J., Jiménez-Gómez, J. M., & Lippman, Z. B. (2017). Variation in the flowering gene SELF PRUNING 5G promotes day-neutrality and early yield in tomato. *Nature Genetics*, *49*(1), 162–168.

- Su, T., Liu, H., Wu, Y., Wang, J., He, F., Li, H., Li, S., Wang, L., Li, L., Cao, J., Lu, Q., Zhao, X., Xiang, H., Lin, C., Lu, S., Liu, B., Kong, F., & Fang, C. (2024). Soybean hypocotyl elongation is regulated by a MYB33-SWEET11/21-GA2ox8c module involving long-distance sucrose transport. *Plant Biotechnology Journal*, *22*(10), 2859–2872.
- Takagi, H., Ito, S., Shim, J. S., Kubota, A., Hempton, A. K., Lee, N., Suzuki, T., Yang, C., Nolan, C. T., Bubb, K. L., Alexandre, C. M., Kurihara, D., Sato, Y., Tada, Y., Kiba, T., Pruneda-Paz, J. L., Queitsch, C., Cuperus, J. T., & Imaizumi, T. (2025). *A florigen-expressing subpopulation of companion cells expresses other small proteins and reveals a nitrogen-sensitive FT repressor*. https://doi.org/10.7554/elife.102529.1
- Takahashi, Y., Teshima, K. M., Yokoi, S., Innan, H., & Shimamoto, K. (2009). Variations in Hd1 proteins, Hd3a promoters, and Ehd1 expression levels contribute to diversity of flowering time in cultivated rice. *Proceedings of the National Academy of Sciences of the United States of America*, *106*(11), 4555–4560.
- Turner, A., Beales, J., Faure, S., Dunford, R. P., & Laurie, D. A. (2005). The pseudo-response regulator Ppd-H1 provides adaptation to photoperiod in barley. *Science (New York, N.Y.)*, *310*(5750), 1031–1034.
- Undurraga, S. F., Press, M. O., Legendre, M., Bujdoso, N., Bale, J., Wang, H., Davis, S. J., Verstrepen, K. J., & Queitsch, C. (2012). Background-dependent effects of polyglutamine variation in the Arabidopsis thaliana gene ELF3. *Proceedings of the National Academy of Sciences of the United States of America*, *109*(47), 19363–19367.
- Xue, W., Xing, Y., Weng, X., Zhao, Y., Tang, W., Wang, L., Zhou, H., Yu, S., Xu, C., Li, X., & Zhang, Q. (2008). Natural variation in Ghd7 is an important regulator of heading date and yield potential in rice. *Nature Genetics*, *40*(6), 761–767.
- Zagotta, M. T., Hicks, K. A., Jacobs, C. I., Young, J. C., Hangarter, R. P., & Meeks-Wagner, D. R. (1996). The Arabidopsis ELF3 gene regulates vegetative photomorphogenesis and the photoperiodic induction of flowering. *The Plant Journal: For Cell and Molecular Biology*, *10*(4), 691–702.
- Zhang, B., Liu, H., Qi, F., Zhang, Z., Li, Q., Han, Z., & Xing, Y. (2019). Genetic interactions among Ghd7, Ghd8, OsPRR37 and Hd1 contribute to large variation in heading date in rice. *Rice (New York, N.Y.)*, *12*(1), 48.
- Zhang, S., Jiao, Z., Liu, L., Wang, K., Zhong, D., Li, S., Zhao, T., Xu, X., & Cui, X. (2018). Enhancer-promoter interaction of SELF PRUNING 5G shapes photoperiod adaptation. *Plant Physiology*, *178*(4), 1631–1642.
- Zhang, Y., Liu, Z., Wang, L., Zheng, S., Xie, J., & Bi, Y. (2010). Sucrose-induced hypocotyl elongation of Arabidopsis seedlings in darkness depends on the presence of gibberellins. *Journal of Plant Physiology*, *167*(14), 1130–1136.
